# Health-related quality of life in stroke patients questionnaire, short version (HRQOLISP-40): validation for its use in Colombia

**DOI:** 10.1186/s12883-016-0770-5

**Published:** 2016-11-28

**Authors:** Yahira Rossini Guzmán Sabogal, Jorge Pla Vidal, Ricardo Sánchez Pedraza, Felipe Ortuño Sánchez-Pedreño, María Catalina Gómez Guevara

**Affiliations:** 1 Psychiatry Department, Universidad de La Sabana, Chía, Colombia; 2 Psychiatry Department, Universidad de Navarra, Pamplona, Spain; 3 Psychiatry Department, Universidad Nacional de Colombia, Bogotá, Colombia; 4 Rehabilitation Department, Clínica Universidad de La Sabana, Chía, Colombia

**Keywords:** Stroke, Quality of life, Measurement instruments, Rehabilitation, HRQOLISP-40, Colombia

## Abstract

**Background:**

The health-related quality of life in stroke patients (HRQOLISP-40, short version) survey was developed in Nigeria and constitutes a 40-item, multidimensional, self-administrated questionnaire. We assessed the validity and reliability of the HRQOLISP-40 Spanish version for stroke patients in Colombia.

**Methods:**

The analysis included factor analysis, confirmatory factor analysis, Rasch analysis, convergent validity, internal consistency (261 stroke patients), test-retest reliability (73 patients assessed at two different times) and sensitivity to change (46 patients assessed before and after a rehabilitation intervention).

**Results:**

We found an 8-domain structure. None of the items had a significant impact on the global alpha value in order to be removed. Lin’s concordance correlation coefficient indicated test-retest reliability (Rho IC: 0.76 to 0.95), suggesting an adequate stability of the instrument. Regarding sensitivity to change differences, they were only significant in the psychological and eco-social domains (*p* <0.05). When comparing SF-36 with HRQOLISP-40, all the correlation coefficients values were significantly different from zero, except those related to vitality. The highest scores were found in the physical and physical functioning domains, with a value of 0.722.

**Conclusions:**

The HRQOLISP-40 scale is valid and reliable for assessing patients’ quality of life after a stroke. Validating quality of life assessment instruments is necessary in order to improve the effectiveness of rehabilitation programs for Colombian stroke patients.

**Electronic supplementary material:**

The online version of this article (doi:10.1186/s12883-016-0770-5) contains supplementary material, which is available to authorized users.

## Background

Stroke is the third leading cause of death and the most frequent cause of disability in adults [1, 2]. One in 17 deaths in the United States is caused by stroke and many victims are left with significant cognitive impairment and decreased quality of life after the event [3]. In Latin America, the incidence rate reported ranges between 0.89 and 1.83/1000, tripling over the age of 60. [4]. According to the World Health Organization (WHO) [5], a stroke is defined as the rapid development of focal or global signs evidencing compromised brain function, with symptoms that can last up to 24 h or more or that can cause death through a vascular cause alone. Its consequences will depend on the size and location of the lesion [3, 6, 7]. The economic and social costs from neurological sequels are high and include health system support strain, function loss in patients and the possibility of patients developing emotional symptoms [8]. Moreover, most studies evidence some of the problems related to measuring the effectiveness of cognitive rehabilitation processes [9]. Given the methodological difficulties found in some studies from different countries and the lack of validated instruments with enough measurement capabilities for different populations, the instruments to be used nowadays must be chosen depending on the research question and considering the specific clinimetric properties from the assessment scale [10–12].

The complete rehabilitation of patients with stroke sequels has sparked wide interest in understanding and assessing the factors that promote a better prognosis in these patients [13], ensuring that they follow proper medical indications [14, 15]. Stroke survivors are at a greater risk of developing emotional symptoms that can interfere with their rehabilitation process and affect their recovery [16, 17]. In addition, issues related to patients’ individual perceptions of status, position in life, value systems, expectations, etc. loom large, requiring individualised psychosocial interventions to be implemented with caution [16, 18, 19].

According to the WHO, quality of life and palliative care involve the prevention and relief of suffering and are carried out through the identification, assessment and treatment of pain and physical problems; it also makes very clear that psycho-social [13] and spiritual aspects [20] are equally important.

According to Barclay & Tate’s prospective study carried out in 2014, stroke patients present a change in the responses observed over time, which they interpret as changes in life priorities according to each individual’s post-stroke adaptation process. This alters the results in the quality of life self-assessment carried out by patients over time [21] and it also becomes another reason to carry out studies assessing the instruments designed to measure quality of life in patients who have had a stroke [22].

Most instruments used to measure patients’ quality of life are generic, however some specific ones exist and they are difficult to compare given that they measure diverse domains. For example, some relate to functionality, which affects the patient’s general perception of life and, consequently, affects his or her quality of life [23, 24]. When measuring quality of life in stroke patients, it is important to remember that very small changes can accumulate over time and these instruments should have the ability to detect them, since effectively measuring post-rehabilitation objectives may depend on it [25].

This study takes into account the social and economic impacts that arise from a stroke and the current argument that it is necessary to provide high quality rehabilitation programs [26]. Evidence suggests that physical therapy conducted during acute rehabilitation of stroke patients reduces costs and improves quality of life adjusted by years. However, there is uncertainty about its long-term cost-effectiveness because of the difficulties highlighted in the small amount of evidence available to date [27].

The purpose of this study is to validate the HRQOLISP-40 instrument, which Dr. Mayowa Ojo Owolabi developed in Nigeria. This scale is novel in that it includes specific questions related to the spiritual component within its domain [28, 29]. The same researcher confirmed his findings in a study published in 2013 [30], which raises the possibility that the spiritual domain influences the prognosis of stroke patients and that therapeutic interventions in this regard might actually reduce the condition’s biographic impact on patients. In fact, the specific proposal based on Dr. Owolabi’s observations purports that just as the physical component is important in the rehabilitation process for stroke patients, so is the implementation of strategies that address the spiritual domain in said interventions, as well as the development of research aimed at ‘healing’ the spirit when it comes to quality of life [30–33].

## Methods

The HRQOLISP-40 scale consists of a section of instructions and 40 items written on a Likert-like scale. The instrument includes the following domains: physical, psycho-emotional, cognitive, eco-social, related to the soul, spiritual, and finally, spiritual interaction. The Likert-like scale has 5 response options for all items. For item 1, the options include, ‘bed bound, chair bound, walks with helpers, walks with aids (frame/tripod) and walks unaided’.

For items 2–4 (physical domain), 1–3 (cognitive domain), 2–4 (eco-social domain), 1–4 (soul domain), 1–3 (spiritual domain) and 1–2 (spiritual interaction), the response options include, ‘not at all, a little, a moderate amount, very much and extremely’.

For items 5–7 (physical domain), 7 (psycho-emotional domain), 4–5 (cognitive domain), 5–7 (eco-social domain), 5–6 (soul domain), 4 (spiritual domain) and 3–4 (spiritual interaction), response options include, ‘very dissatisfied, dissatisfied, neither satisfied nor dissatisfied, satisfied and very satisfied’.

For items 1–6 (eco-emotional domain), response options include, ‘not at all/never, a little/seldom, moderately/quite often, mostly/very often and completely/always’.

For item 1 (eco-social domain), response options include, ‘fully dependent, require substantial help, require minimal help, require no help, but not back to work, and back to work’.

Scores for each domain were generated with the Likert method, which means that the response to the item was added without weighting or standardising it, thus facilitating its interpretation and inter-individual comparison. Domain scores were transformed to a maximum score of 100 for each one. In order to grade them, items whose number is accompanied by a comma or apostrophe (') were scored as negative (i.e., register as −1). Domain scores (the sum of all item scores) were generated in such a way that, as discussed above, the maximum score can be transformed to 100 [29].

Colombian rehabilitation teams require more and better research in order to determine their rehabilitation activities’ effectiveness and to clarify the interactions between different areas within the rehabilitation process. In addition, measuring changes and improving the quality of care for patients based on measurable attributes such as quality of life requires the validation of the instruments designed for this purpose.

It is crucial that the instruments designed to measure quality of life involve aspects such as spirituality, given that their importance has been widely established for chronic and terminal illnesses [34, 35]. Since there are measuring instruments designed to better understand spirituality, we decided to use a scale that involves this aspect.

The HRQOLISP-40 scale was used in the treatment of 261 patients who visited the rehabilitation unit at the Universidad de La Sabana Hospital, the vascular neurology consultation service at the Kennedy University Hospital and the emergency and hospitalisation service at the Hernando Moncaleano Perdomo University Hospital between May 2013 and May 2015, with a preliminary stage of cultural adaptation between January and March 2013 [36, 37]. This sample was used to carry out exploratory factor analysis, confirmatory analysis, model measurement using the item response theory and an assessment of internal consistency and convergent validity (for this purpose, the quality of life scale Sf −36 [38] was used at the same time in 73 patients).

The test-retest reliability assessment was obtained by implementing the instrument in two instances on 73 patients; the average time-lapse between both measurements was 11.2 days (SD = 6.6 days). In order to assess sensitivity to change, the instrument was implemented in two instances on a total of 46 patients (before and after an intervention rehabilitation session) in accordance with each patient’s condition.

Patients included in groups for assessing the test-retest reliability and sensitivity to change came from a subset of the total 261 patients.

For each of the scale validation components, sample size calculations were carried out using PASS® software.

### Statistical analyses

For psychometric related components correlation coefficients were taken as those moderate correlation values greater than 0.50 and as high values those over 0.7 [39]. They were considered as Cronbach’s alpha values of those larger than 0.7 [40].

For the content validity study, we used an exploratory factor analysis that allowed us to evaluate the latent variables structure reflected by the construct when it was measured with the HRQOLISP-40 scale in the Colombian patient sample used. For this analysis, we used the principal factor method, estimating a minimum sample size of 250 patients [35]. In addition, a confirmatory factor analysis method was carried out using the structural equations method. Taking into account the ordinal nature of the item scores in the Likert-like scale, we used estimation methods that handled polychoric correlations and asymptotic covariance matrices [41]. These matrices were generated using STATA 13® software. The matrix factorability was defined with the Bartlett’s sphericity test and the Kaiser Maeyer-Olkin test. Scree plots and the number of *eigenvalues* greater than one were used as the criteria to select the number of domains to analyse; likewise, factor loading criteria greater than 0.3 was also used to evaluate the domains’ conformation [42]. Both orthogonal and oblique rotations were applied to find the most suitable factor loading option. The interpretability of domains in each factor was applied in order to select the best factorial structure. For the structural equations component, we took the following criteria to assess the model adjustment: *χ*2 ratio out of the degrees of freedom (*χ*2 / df) < 3, Root Mean Square Error of Approximation (RMSEA) <0.08, Tucker-Lewis Index (TLI) and Comparative fit Index (CFI) > 0.98. In addition, lower values from our calculation of the Bayesian Information Criterion (BIC) and the Akaike Information Criterion (AIC) suggest a better adjustment.

A Rasch analysis was carried out to evaluate person and item reliability; these reliability indices are analogous to Cronbach’s alpha values (which range between 0 and 1). Within this analysis, we also assessed the separation indices for persons and items (values higher than 2 were considered as good separation indicators, so this value was used as the cut-off point), as well as item-fit statistics (INFIT and OUTFIT item tests), with the aim of determining construct homogeneity and item redundancy. These analyses were carried out using Winsteps® software and a partial credit model for polytomous data.

Cronbach’s alpha coefficients for the entire scale, for each of the domains, and for the scale with the removal of each one of the items were calculated in order to assess internal consistency. For this purpose, we estimated that a sample size of 101 patients allows for the detection of a difference between an alpha coefficient of 0.7 for the null hypothesis and 0.8 for the alternative, having a power of 80% and a significance level of 5%. On the other hand, for the sensitivity to change test, we calculated a sample size of 40 patients and assumed a type I error of 0.05, a power of 0.80, a difference of at least 10 points in the scale score between the different measurement points taken before and after a rehabilitation intervention. This calculation takes into account the non-independence of mean measurements before and after an intervention, considering the use of paired t-tests. To calculate the test-retest reliability, the scale was applied in two instances separated by a period of between 7 and 15 days; likewise, we assumed a type I error of 0.05, a power of 0.8, a Lin’s correlation and concordance coefficient value [34] equal to 0.92 for the alternative hypothesis and equal to 0.86 for the null hypothesis, allowing us to determine a sample size of 70 patients.

To calculate the sample size for the convergent criterion validity component, we assumed a type I error of 0.05, a power of 0.8, a Lin’s correlation and concordance coefficient value [34] equal to 0.2 for the null hypothesis and equal to 0.5 for the alternate hypothesis. The outcome allowed us to determine a sample size of 70 patients.

The sample size calculation that corresponds to the item response theory procedures (Rasch model) took into account the recommendation of including at least 250 observations when using Likert-like scales [35].

The study was carried out following the Declaration of Helsinki guidelines and was approved by the Universidad de La Sabana ethics committee, according to Minute 246 on March 15, 2013 and all patients signed an informed consent at each health institution involved in the study.

## Results

Altogether, 261 instruments were applied, from which 118 (45.2%) were obtained from the Universidad de La Sabana University Hospital, 91 (34.9%) from the Kennedy University Hospital and 52 (19.9%) from the Hernando Moncaleano Perdomo University Hospital in Neiva. 152 patients (58.2%) were men, which was the predominant gender in all the 3 sites sampled. The average age (standard deviation) for each site was 56.02 (16.80), 65.44 (12.70) and 71.54 (10.01), respectively.

Regarding the type of stroke found in patients, 84.36% corresponded to ischemic strokes (83.13% of these were thrombotic), 12.26% to intraparenchymal haemorrhages and 3.10% to subarachnoid haemorrhages. As for the religious aspect, 214 patients (82%) reported themselves to be Catholic, 26 (10%) Christian, 12 (4.6%) said they had other religions and 9 (3.4%) reported having no religion.

Instrument implementation took 19.16 min on average per patient, the minimum time was 9 min and, for 3 patients, it took 40 min.

The highest mean measurement score for each one of the items was 4.5 and corresponded to the item d1_1 ‘Mobility’, while the items d1_6 ‘How satisfied are you with your ability to work?’ and d7_2 ‘To what extent do you discuss aspects of your faith/religion with people of the same religion/interest/faith, in order to strengthen your individual purpose?’ obtained the lowest scores.

The mean scores (standard deviation) obtained in each of the domains included in the scale were as follows: physical, mean = 69 (SD = 17.6), psychological, mean = 66 (SD = 17), cognitive, mean = 65 (SD = 18.9), eco-social, mean = 71 (SD = 13.8), related to the soul, mean = 74 (SD = 15), spiritual, mean = 73 (SD = 16.6), and spiritual interaction, mean = 70 (SD = 14.6).

### Exploratory factor analysis

An exploratory factor analysis was carried out through a principal component factors method, using the data obtained from 261 patients. Based on the criteria described in the above-mentioned methodology, the optimal number of domains was eight.

The resulting factor structure can be observed in Table 1, according to the proposed number of domains. This structure corresponds to an orthogonal rotation (varimax).

**Table 1:** Domain structure according to the proposed number of domains for the HRQOLISP-40 scale

Item	F1	F2	F3	F4	F5	F6	F7	F8	u2^a^
d6_3 To what extent do you understand your religion or faith?	0.78								0.27
d5_4 To what extent do you practice your religion or faith?	0.76								0.36
d6_1 To what extent do you understand God?	0.74								0.32
d6_2 To what extent are you guided or motivated by God in your daily life?	0.69								0.26
d7_2 To what extent do you discuss aspects of your faith/religion with other people?	0.64								0.45
d7_1 How close do you consider yourself to God or your religious beliefs?	0.64								0.32
d1_2 To what extent or with how much difficulty do you use your hands?		0.76							0.34
d1_1 Mobility		0.76							0.27
d1_3 How much difficulty do you have sitting/standing without losing your balance?		0.76							0.27
d4_1 Daily activities (eating, bathing, toileting, etc.)		0.73							0.21
d1_4 To what extent do you think pain, malaise and/or loss of sensation, limits your ability?		0.55							0.44
d4_4 How much access do you have to transportation?		0.48							0.43
d3_3 To what extent are you able to communicate?			0.82						0.23
d3_4 How satisfied are you with your ability to communicate?			0.78						0.25
d3_5 How satisfied are you with your ability to think and learn?			0.66						0.37
d3_2 How accessible is the information that you need for your day-to-day life?			0.64						0.36
d3_1 How good is your ability to concentrate?			0.48						0.51
d2_3 To what extent are you able to accept your physical appearance?			0.47						0.46
d7_3 How satisfied are you with your relationship with God or your religious beliefs?				0.78					0.25
d7_4 How satisfied are you with your efforts to develop your faith/religion?				0.7					0.38
d5_5 How satisfied are you with your faith in God?				0.68					0.3
d6_4 How satisfied are you with the divine guidance in your life?				0.59					0.4
d5_2 To what extent do you believe you have a purpose in life?					0.78				0.21
d5_3 How interested are you in fulfilling your life purpose?					0.75				0.25
d5_1 How much self-confidence do you have?					0.62				0.39
d5_6 How satisfied do you feel about yourself?					0.54				0.36
d4_2 How much respect do you receive from others?					0.46				0.47
d1_7 How satisfied are you with your sex life?						0.7			0.44
d1_6 How satisfied are you with your ability to work?						0.63			0.34
d2_4 How much do you enjoy your job?						0.55			0.38
d1_5 How satisfied are you with your ability to perform everyday activities?						0.53			0.36
d4_3 How able are you to manage your home and domestic roles?						0.42			0.38
d2_1 How often do you have feelings such as sadness, anger, etc.?							0.76		0.38
d2_7 How satisfied are you with your feelings?							0.6		0.4
d2_2 Do you feel you have enough energy to face each day?							0.52		0.4
d2_5 How often do you laugh?							0.46		0.43
d2_6 To what extent do you enjoy your free time?							0.42		0.42
d4_6 How satisfied are you with the support you receive from your friends?								0.73	0.37
d4_7 How satisfied are you with your access to health services?								0.49	0.55
d4_5 How satisfied are you with your inter-personal relationships?								0.48	0.45

^a^ Uniqueness. *The table only shows the factor loadings > or = 0.3*

The variance proportion for each of the domains resulted as follows:

Domain 1: 0.10, Domain 2: 0.10, Domain 3: 0.10, Domain 4: 0.08, Domain 5: 0.08, Domain 6: 0.07, Domain 7: 0.06 and Domain 8: 0.05. The total variance found in the eight domains was 64%.

Given their factors, structure and characteristics, the domains were identified as follows: spiritual or belief in God, cognitive/communicative, physical ability, satisfaction with spiritual aspects, self-perception/transcendental life, psycho-emotional, eco-social and satisfaction with one’s capabilities.

### Confirmatory factor analysis

Table 2 shows the goodness of fit indices corresponding to confirmatory analyses for both models.

**Table 2:** Goodness of fit corresponding to three factorial models

	Complete model	Adjusted model to factor solution	Adjusted model for modification indices
chi2_ms (783)	3029.686	2497.779	1951.519
RMSEA	0,110	0,096	0,08
AIC	28077.285	27545.378	26835.118
BIC	28503.64	27971.733	26970.131
CFI	0.583	0,70	0,79
TLI	0.56	0,66	0,76

*RMSEA* Root mean squared error of approximation, *AI*C Akaike criterion information

*BIC* Bayesian criterion information, *CFI*Comparative fit index, *TLI* Tucker-Lewis inde*x*

**Fig. 1:**
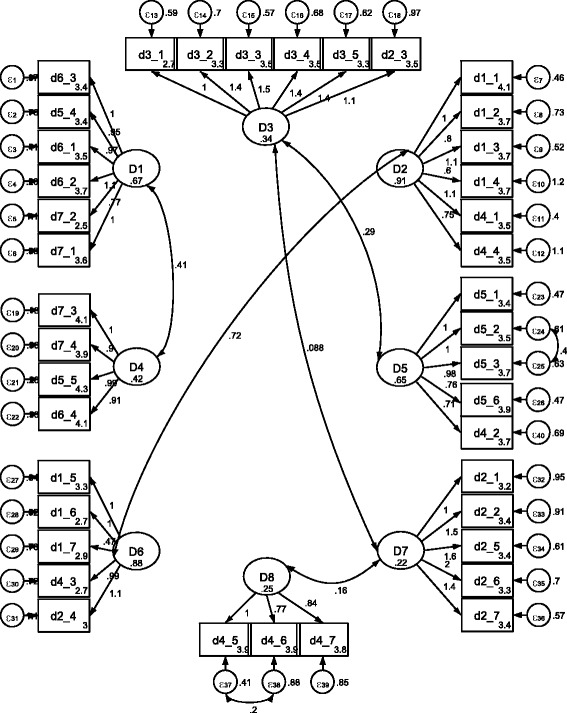
Factor model structure including covariance pathways suggested by modification indices. *D1: Physical domain, D2: Psycho-emotional domain, D3: Cognitive domain, D4: Eco-social domain, D5: Soul domain, D6: Spiritual domain, D7: Spiritual interaction domain. Source: Data from the study results*

The best-fit model incorporates the items according to the factor analysis ordering and the modification indices. Figure 1 shows the adjusted model structure (it also includes the covariance pathways suggested by modification indices).

### Item response theory (Rasch), scale validation

Analyses were carried out using Rasch models for polytomous data. Information on the overall model adjustment is presented in Table 3, where SD has ZSTD values for items greater than 2, suggesting a poor adjustment for these items.

**Table 3:** Global adjustment measures for the instrument

	Infit		Outfit		Separation	Reliability
	*MNSQ*	*ZSTD*	*MNSQ*	*ZSTD*		
Persons						
Measurement	1.02	−0.2	1.02	−0.2	3.69	0.93
SD	0.46	2.1	0.47	2.1		
Items						
Measurement	1.00	−0.2	1.02	0.1	6.64	0.98
SD	0.22	2.6	0.23	2.5		

*MNSQ* mean-squar., *ZSTD* standardised fit statistics

Reliability and separation of people and items indices for each of the seven domains are presented in Table 4.

**Table 4:** Indices for people-items separation for scale domains

Domains		Reliability index	Separation index
D1 Physical	Person	0.79	1.92
	Item	0.99	8.42
D2 Psycho-emotional	Person	0.88	1.89
	Item	0.89	2.83
D3 Cognitive	Person	0.81	2.04
	Item	0.98	6.41
D4 Eco-social	Person	0.72	1.6
	Item	0.98	6.43
D5 Soul	Person	0.74	1.71
	Item	0.98	6.79
D6 Spiritual	Person	0.79	1.94
	Item	0.98	7.97
D7 Spiritual interaction	Person	0.67	1.43
	Item	0.99	13.44

The reliability values are > 0.89 for items and > 0.67 for people. The fact that separation indices are much better for items than for people suggests restricted attribute amplitude (quality of life) in this sample of patients.

Table 5 shows adjustment statistics by weighted information criterion (infit) and by extreme values or outlier criterion (outfit) on the scale items. Items with infit or outfit values > 1.4 and associated ZSTD values > 2.0 are considered to have poor adjustment; in this sense, the items’ redundancy is suggested by infit-outfit values to be < 0.6.

**Table 5:** Adjustment statistics for items

		Infit		Outfit	
Item		MNSQ	ZSTD	MNSQ	ZSTD
d1_1	Mobility	1.26	2.7	1.16	1.6
d1_2	To what extent or with how much difficulty do you use your hands to grasp objects, turn the doorknob, use silverware, write, open a jar or gallon tank or lift heavy objects?	1.25	2.8	1.36	3.7
d1_3	How much difficulty do you have sitting/standing without losing your balance?	1.2	2.3	1.12	1.4
d1_4	To what extent do you think pain, malaise and/or loss of sensation limits your ability to do what you need to do?	1.56	5.8	1.62	6
d1_5	How satisfied are you with your ability to perform everyday activities (eating, bathing, toileting, dressing, grooming, etc.)?	0.96	−0.5	0.95	−0.6
d1_6	How satisfied are you with your ability to work?	0.99	−0.2	0.97	−0.4
d1_7	How satisfied are you with your sex life?	0.89	−1.4	1.03	0.4
d2_1	How often do you have feelings such as sadness, anger, desperation, anxiety, depression and/or fear?	1.14	1.8	1.24	2.8
d2_2	Do you feel you have enough energy to face each day?	0.97	−0.3	0.99	−0.1
d2_3	To what extent are you able to accept your physical appearance?	1.08	1	1.06	0.7
d2_4	How much do you enjoy your job?	1.43	4.9	1.4	4.5
d2_5	How often do you laugh?	0.87	−1.6	0.88	−1.4
d2_6	To what extent do you enjoy your free time?	1.05	0.7	1.08	0.9
d2_7	How satisfied are you with your feelings?	0.79	−2.9	0.79	−2.7
d3_1	How good is your ability to concentrate?	0.68	−4.5	0.68	−4.4
d3_2	How accessible is the information that you need for your day-to-day life?	0.98	−0.2	0.97	−0.4
d3_3	To what extent are you able to communicate?	1.07	0.9	1.07	0.8
d3_4	How satisfied are you with your ability to communicate?	1.13	1.6	1.11	1.3
d3_5	How satisfied are you with your ability to think and learn?	0.94	−0.7	0.91	−1.2
d4_1	Daily activities (eating, bathing, toileting, etc.)	0.91	−1.1	0.88	−1.5
d4_2	How much respect do you receive from others?	0.89	−1.4	0.94	−0.6
d4_3	How able are you to manage your home and domestic roles?	0.89	−1.4	0.88	−1.5
d4_4	How much access do you have to transportation?	1.19	2.2	1.16	1.9
d4_5	How satisfied are you with your inter-personal relationships?	0.65	−4.7	0.71	−3.6
d4_6	How satisfied are you with the support you receive from your friends?	1.27	2.9	1.47	4.5
d4_7	How satisfied are you with your access to health services?	1.13	1.5	1.24	2.5
d5_1	How much self-confidence do you have?	0.72	−3.9	0.73	−3.5
d5_2	To what extent do you believe you have a purpose in life?	0.87	−1.7	0.86	−1.8
d5_3	How interested are you in fulfilling your life purpose?	0.93	−0.8	0.93	−0.8
d5_4	To what extent do you practice your religion or faith?	1.33	3.8	1.41	4.5
d5_5	How satisfied are you with your faith in God?	0.77	−2.5	0.77	−2.3
d5_6	How satisfied do you feel with yourself?	0.72	−3.5	0.72	−3.3
d6_1	To what extent do you understand God?	0.93	−0.9	0.98	−0.2
d6_2	To what extent are you guided or motivated by God in your daily life?	0.88	−1.5	0.87	−1.6
d6_3	To what extent do you understand your religion or faith?	0.92	−1	1	0
d6_4	How satisfied are you with the divine guidance in your life?	0.73	−3.2	0.72	−3
d7_1	How close do you consider yourself to God or your religious beliefs?	0.88	−1.5	0.93	−0.8
d7_2	To what extent do you discuss aspects of your faith/religion with other people of the same faith/interest/religion with the objective of strengthening your individual purpose?	1.43	4.6	1.4	4.3
d7_3	How satisfied are you with your relationship with God or your religious beliefs?	0.68	−3.8	0.71	−3.2
d7_4	How satisfied are you with your efforts to develop your faith/religion?	0.84	−1.9	0.96	−0.4

*MNSQ* mean-squar., *ZSTD* standardised fit statistics

We can see that item d1_4 ‘To what extent do you think pain, malaise and/or loss of sensation, limits your ability to do what you need to do?’ demonstrates poor adjustment. Other items that suggest poor adjustment values are: d4_6 ‘How satisfied are you with the support you receive from your friends?’, d2_4 ‘How much do you enjoy your job?’ and d7_2 ‘To what extent do you discuss aspects of your faith/religion with other people of the same faith/interest/religion with the objective of strengthening your individual purpose?’ The analysis does not suggest the presence of redundant items.

The mean scores presented in Table 6, which are an average of the differences found between the skill values and item difficulty, show an increasing monotonic trend in each of the domains. This suggests that patients with a higher quality of life tend to score each item within the different categories higher. The adjustment values by weighted information criterion (infit) and by outlier criterion (outfit) are within the range of 0.6-1.4.

**Table 6:** Average measurements for each domain category

Domain and category (item)		Average *measurement*	Infit *MNSQ*	Outfit *MNSQ*
*D1 Physical*	1	−1.17	1.31	1.32
	2	−0.52	0.83	0.81
	3	0.16	1.10	1.29
	4	1.04	0.79	0.80
	5	1.92	0.97	1.01
*D2 Psycho-emotional*	1	−0.92	1.14	1.10
	2	−0.49	0.94	0.98
	3	0.22	0.97	0.95
	4	0.88	0.83	0.83
	5	1.53	1.07	1.06
*D3 Cognitive*	1	−2.31	1.40	1.17
	2	−0.93	0.95	0.97
	3	0.42	0.92	0.96
	4	1.51	0.84	0.80
	5	2.52	1.01	1.02
*D4 Eco-social*	1	−0.92	0.99	1.14
	2	−0.24	0.94	0.99
	3	0.31	0.87	0.87
	4	1.00	0.88	1.00
	5	1.73	1.11	1.08
*D5 Soul*	1	−1.08	1.33	1.32
	2	−0.46	1.03	1.08
	3	0.46	0.88	0.89
	4	1.50	0.90	0.95
	5	2.63	1.01	1.00
*D6 Spiritual*	1	−4.64	1.10	1.07
	2	−1.74	1.08	1.10
	3	0.65	0.79	0.79
	4	2.99	0.82	1.14
	5	4.52	1.25	1.23
*D7 Spiritual Interaction*	1	−2.59	0.95	0.96
	2	−1.36	0.89	1.03
	3	0.06	0.78	0.74
	4	1.73	0.78	1.30
	5	3.12	1.33	1.18

**Fig. 2:**
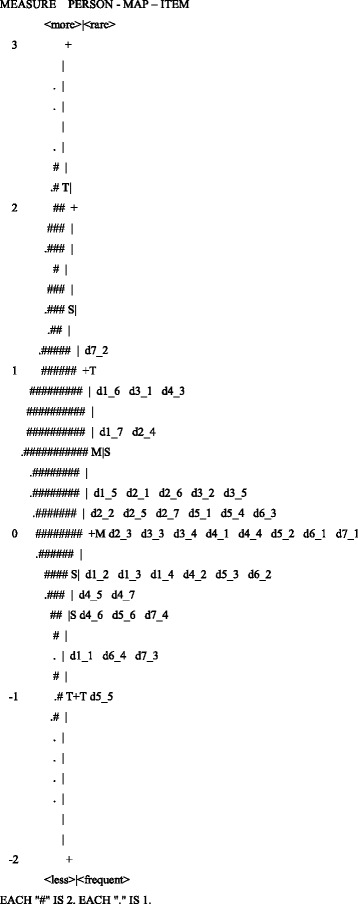
Item-person map. *Each “#” means two patients and each “.” means one patient. Source: Data from the study results*

In Fig. 2, the higher up a patient is on the vertical scale, the better quality of life he or she experiences. As we can see, there is a group of 45 patients with high attribute levels that are not covered by the scale. The figure also shows that the means for items and persons (patients) differ by about 0.5 logits, with the patient average higher. This suggests that the latent attribute (quality of life) that this group experiences is greater than what the scale can measure, which corresponds to a ceiling effect. In addition, item, d7_2 ‘To what extent do you discuss aspects of your faith/religion with other people of the same faith/interest/religion with the objective of strengthening your individual purpose?’ does not seem to properly measure the attribute because its distance to the mean is greater than two standard deviations (the same item showed poor adjustment properties). Item d5_5 ‘How satisfied are you with your faith in God?’ is not very useful for measuring the attribute’s intensity because even patients with low quality of life tend to give high responses. The most representative items for the attribute are d2_3 ‘To what extent are you able to accept your physical appearance?’, d3_3 ‘To what extent are you able to communicate?’, d3_4 ‘How satisfied are you with your ability to communicate?’, d4_1 ‘Activities of daily living (eating, bathing, toileting, etc.)’, d4_4 ‘How much access do you have to transportation?’, d5_2 ‘To what extent do you believe you have a purpose in life?’, d6_1 ‘To what extent do you understand God?’ and d7_1 ‘How close do you consider yourself to God or your religious beliefs?’

### Internal consistency

The alpha coefficient value for the total scale was 0.94. We found that none of the items greatly increased the overall alpha value. The alpha coefficient for each one of the domains had values above 0.72, with spiritual interaction as the lowest value (0.72) and spiritual domain as the highest value (0.86).

### Concurrent validity criterion

For this analysis component, measurements were carried out and assessed simultaneously in 82 patients using the HRQOLISP-40 and SF-36 scales. Coefficient values were calculated based on the correlation between the total domain scores for both scales. The results are presented in Table 7.

**Table 7:** Total domain scores for the HRQOLISP-40 and SF-36 scales

	Physical	Psycho-emotional	Cognitive	Eco-social	Soul	Spiritual	Spiritual Interaction
Physical functioning	*0.722*	0.460	*0.546*	*0.614*	*0.512*	0.267	0.320
Physical role	*0.601*	*0.529*	0.493	0.478	0.446	0.315	0.332
Corporal pain	0.474	0.279	0.250	0.422	0.282	0.370	0.355
General health	*0.581*	*0.610*	*0.515*	*0.533*	0.486	0.374	0.463
Vitality	0.153	0.188	0.147	0.115	0.246	0.186	0.235
Social functioning	0.415	0.485	0.487	0.447	0.308	0.238	0.279
Emotional role	0.409	*0.613*	0.536	0.467	0.415	0.178	0.270
Mental health	*0.594*	*0.582*	*0.515*	*0.506*	0.455	0.333	0.363

*Rows* FS-36, *Columns* HRQOLISP-40

Except for some values, all the correlation coefficient values are significantly different from zero. Physical and physical functioning domains had the highest scores, while the lowest scores were related to vitality.

### Test-retest reliability

Repeated measurements were carried out in 73 patients using the scale. Means for each domain correspond to each one of the measurements and are presented in Table 8. The concordance-correlation coefficients were between 0.76 (cognitive domain) and 0.95 (spiritual domain).

**Table 8:** Correlation concordance coefficients

Domain	Lin’s Rho	95% CI
Physical	0,93	0,89 - 0,96
Psycho-emotional	0,93	0,89 - 0,96
Cognitive	0,76	0,67 - 0,86
Eco-social	0,93	0,90 - 0,96
Soul	0,94	0,91 - 0,96
Spiritual	0,95	0,93 - 0,97
Spiritual interaction	0,94	0,92 - 0,97

For each domain, means were compared between both measurement instances using paired t-tests. The differences between both measurements taken were not significant.

### Sensitivity to change

The scale was applied to 46 patients before and after an intervention based on an institutional rehabilitation protocol that was tailored to each patient’s condition; the time between both evaluations was at least two months and up to a maximum of six months.

Scores in each domain according to both measurement times are presented in Table 9.

**Table 9:** Measurements before and after rehabilitation intervention

Domain	Measurement 1		Measurement 2	
	Mean	SD	Mean	SD
D1 Physical	69	2.1	72.2	2.2
D2 Psycho-emotional^b^	68.2	2.6	72.8	2.5
D3 Cognitive	69.8	2.5	73	2
D4 Eco-social^a^	73.1	2	77.1	1.5
D5 Soul	76.9	2.2	77	2
D6 Spiritual	73.6	2.8	75.4	2.3
D7 Spiritual interaction	73	2.2	70.9	2

^a^ t (45) = −2.62, *p* = 0.011, ^b^ t (45) = −2.27, *p* = 0.027

The measurements before and after an intervention were compared using paired t-tests. Although an overall increase in quality of life levels after the intervention is evidenced, the differences were only significant in the psycho-emotional and eco-social domains (*p* <0.05).

## Discussion

The HRQOLISP-40 instrument demonstrates good validity and reliability and is capable of measuring some changes after a rehabilitative intervention with patients, but its sensitivity to change analyses suggests the instrument has limited sensitivity. Both its timeframe and easy scoring process facilitates its implementation in daily clinical practice.

One of the instrument’s strengths identified in this study corresponds to the fact that, in addition to the classic domains, it incorporates the assessment of other human dimensions that are important for patients’ quality of life. To our knowledge, this is the first study using Rasch models for the evaluation of scale properties.

When comparing the average scores for each domain in the Colombian population sample by implementing the original scale used in Berlin and Ibadan, we found that the scale behaved similarly in other populations studied. In Ibadan, the only score below 70 was the psychological domain, while others were between 71 and 83.5 (corresponding to the spiritual domain); in Berlin, the lowest scores were obtained in the spiritual domain and the spiritual interaction scores, which were 45.3 and 46.8 respectively, while other values ranged between 63 and 75.9 (corresponding to the cognitive domain). Data in our study shows that the lowest average was 65 for the cognitive domain and the highest averages were 74 for the soul domain and 73 for the spiritual domain, respectively. Overall, everything related to the spiritual domain weighs more in Ibadan and less in Berlin both for stroke patients and the control groups [29].

In Colombia, mean values varied little between domains when compared to the other two populations and the overall values obtained represent an average between both since the soul and spiritual domains obtained the highest scores. This fact likely highlights the importance of these domains for the patients assessed in this sample, thus reflecting what quality of life means to them. It is also necessary to question why in Colombia the cognitive domain value was the lowest, showing a mean difference of almost 10 points in comparison with the other two values obtained. As a possible interpretation, this result suggests that the Colombian population sample feels more deficient in the cognitive domain or that patients desire an improvement in this area. It also implies the need to improve the cognitive rehabilitation approach using personalised, and even multimodal, strategies tailored to the needs of each patient, applying them more vigorously when necessary and, in any case, with an aim towards meeting the expectations of patients and their families when possible [43, 44].

As for internal consistency, the present study shows a value of 0.94, which suggests that the structure is too homogeneous and that no redundant items were detected. During the validation of the original scale, overall values in Ibadan and Berlin were 0.86 and 0.76, respectively. The lowest alpha coefficient value found was for the spiritual interaction domain (0.72) and the highest value found was for the spiritual domain (0.86), which is consistent with the original study. Nevertheless, the spiritual interaction domain seems to have a less homogeneous structure in the Colombian sample; generally, people tend not to discuss many aspects of their faith or religion and they have some difficulty rating their satisfaction with their efforts to approach or develop this aspect.

The exploratory factor analysis reveals a domain structure that is consistent with the domain organisation proposed by the original scale developers. However, we found the optimal number of domains to be eight and we maintained the following domains: spiritual or belief in God, cognitive/communicative, spiritual interaction, psycho-emotional, eco-social, physical ability and satisfaction with the ability to carry out activities. This implies that, in Colombia, the physical component is divided into two in comparison with the originally proposed assessment; one part herein is related to the implementation of activities and autonomy and the other is related to satisfaction with the ability to carry out activities.

Regarding the spiritual component, in the original scale, the ‘related to the soul’ domain contemplates aspects from both the spiritual domain and what might be called self-perception and transcendental experience; for example, in this analysis, the item ‘To what extent do you practice your religion or faith?’ is most often associated with specific spiritual items and is here referred to as the spiritual domain or belief in God. In addition, this can be seen in the physical domain in that the item ‘To what extent do you have access to transportation?’ is associated with physical domain. The response to this question can be interpreted as the fact that the patient relates this item to physical capacity, rather than to the availability of transportation as such. The other physical domain items correspond qualitatively to the original model.

The satisfaction with spiritual aspects domain clearly groups together items that are consistent with these concepts. Regarding the soul domain, which here is referred to as self-perception and transcendental experience, grouped the following items: ‘To what extent do you believe you have a purpose in life?’, ‘How interested are you in fulfilling your life purpose?’, ‘How satisfied are you with yourself?’, and ‘How much respect do you receive from others?’. The latter item comes from the eco-social domain in the original scale and thus gives this domain a broader meaning, while still measuring the appropriate aspects, and, therefore, complements the self-perception and transcendental domain. This item can be seen as a central element given the fact that, despite the disability a stroke causes, it is still possible to work towards one’s life goals.

In general, the exploratory analysis’s adjustment represents a better adjustment model than the theoretical model.

Regarding concurrent validity, we found good correlation levels between the physical domains of both scales, in which the lowest values were related to the spiritual domain. This result is obvious since the SF-36 does not include this domain within its domain areas [38]. The correlation coefficient values between SF-36 and the HRQOLISP-40 related to the emotional, spiritual and soul domains reached the lowest values between the concordant dimensions of both scales. The SF-36 vitality domain showed lower correlation values regarding the HRQOLISP-40 domains; moreover, most of the values were not significantly different from zero.

When evaluating repeatability within the different domains, the range was from 0.76 (cognitive domain) to 0.95 (spiritual domain). The overall reliability of the instrument measured by repeated applications suggests an adequate stability.

The Rasch analysis reveals that, in general, the instrument shows adequate psychometric properties and specifically indicates an item that does not indicate good adjustment: D1-4 ‘How much do you think pain, malaise and/or loss of sensation limit your ability to do what you need to do?’ Given that pain and other discomforts are part of questions or items covered by quality of life scales, it is possible for the patient to misinterpret the question and it should, therefore, be explained more clearly. Any of the existing items could potentially be replaced if they are found to measure an attribute other than quality of life, thereby not contributing to the scale subject. This seems obvious in item d5_5, ‘How satisfied are you with your faith in God?’, which most people responded to with high scores, despite other indicators of low quality of life.

## Conclusions

The health-related quality of life in stroke patients (HRQOLISP-40, 40-item scale version) is valid and reliable for assessing the quality of life in stroke patients; however, some adjustments are required in order to improve psychometric properties for the Colombian population.

Rasch analysis suggests poor adjustment of some scale items and a model that favours adjustment in the Colombian population.

In the sample analysed, patients seem to have a higher quality of life than the instrument can measure. To overcome this difficulty, it is necessary to incorporate additional items from, for example, an initial qualitative approach to patients with high levels of the attribute. Another possibility for incorporating additional items is by applying the instrument to patients with a lower quality of life and using a larger sample that could also come from other health institutions with different characteristics.

The most representative items of the attribute include d2_3 ‘To what extent are you able to accept your physical appearance?’, d3_3 ‘To what extent are you able to communicate?’, d3_4 ‘How satisfied are you with your ability to communicate?’, d4_1 ‘Activities of daily living (eating, bathing, toileting, etc.)’, d4_4 ‘To what extent do you have access to transportation?’, d5_2 ‘To what extent do you think you have a purpose in life?’, d6_1 ‘To what extent do you understand God?’, and d7_1 ‘To what extent do you consider yourself closer to God or your religious beliefs?’

Item d5_5 ‘To what extent are you satisfied with your faith in God?’ is not very useful for measuring the attribute’s intensity (even patients with a low quality of life tend to give high responses). Thus, this item could be withdrawn.

In addition to the above, the instrument’s utility is favourable because it can be applied in a timely manner and the scoring system presents very little difficulty.

## Additional file

Additional file 1: Data base HRQOLISP-40 Colombia. Description of data: Acces date base. (ACCDB 1828 kb)

## Abbreviations

AIC: Akaike information criterion
BIC: Bayesian information criterion
CFI: Comparative fit index
RMSEA: Root mean square error of approximation
SD: Standard deviation
Sf-36: 36-questionnaire (short form)
TLI: Tucker -lewis index
WHO: World Health Organization
